# Data on characterization of glass biochips and validation of the label-free biosensor for detection of autoantibodies in human serum

**DOI:** 10.1016/j.dib.2020.105648

**Published:** 2020-04-30

**Authors:** A.V. Pushkarev, A.V. Orlov, S.L. Znoyko, D.O. Novichikhin, V.A. Bragina, A.A. Sizikov, E. Alipour, H. Ghourchian, A.I. Nikitin, G.M. Sorokin, B.G. Gorshkov, P.I. Nikitin

**Affiliations:** a Prokhorov General Physics Institute of the Russian Academy of Sciences, 38 Vavilov St., Moscow, 119991, Russia; b Moscow Institute of Physics and Technology, 9 Institutskii per., Dolgoprudny, Moscow Region, 141700, Russia; c Institute of Biochemistry and Biophysics (IBB), University of Tehran, P.O. Box 13145-1384, Tehran, Iran; d Volga branch of Moscow Automobile and Road State Technical University, Cheboksary 428000, Russia; e Chuvash State University, 15 Moskovskij Ave., Cheboksary, 428015, Russia

**Keywords:** autoantibodies, detection in human serum, native kinetics, label-free detection, multiplex sensing, diagnostics of autoimmune disorders

## Abstract

The data represent in-depth characterization of a novel method for highly sensitive simultaneous measuring in human serum of both critical parameters of autoantibodies: concentration and native kinetics. The latter refers to autoantibody interaction with free, not immobilized, antigen. The method and related biosensors are based on the spectral-correlation and spectral-phase interferometry. The data cover: multi-factor optimization and quantitative characterization of the developed affordable single-used biochips, including X-ray photoelectron spectroscopy (XPS) control of chemical modifications of the surface during fabrication; antibody screening; optimization and verification of protocols for label-free biosensing in human serum; mathematical model for fitting experimental data and calculation of kinetic constants of interaction of autoantibodies with free antigen; comprehensive verification of the method specificity; correlation between the data obtained with the developed biosensor and with enzyme linked immunosorbent assay (ELISA); comparison of analytical characteristics of the developed biosensor with the most advanced label-based methods. The data importance is confirmed by a companion paper (DOI 10.1016/j.bios.2020.112187), which shows that the combination of mentioned autoantibody parameters is promising for more accurate criteria for early diagnostics and efficient therapy of autoimmune disorders. The obtained data can be used in development of a wide range of biosensors, both label-free and based on various labels.

Specifications Table

**Table:** 

**Subject**	Analytical chemistry
**Specific subject area**	Simultaneous measuring both critical parameters of several autoantibodies in human serum: kinetics of autoantibody interaction with non-immobilized antigens and concentration

**Type of data**	Tables and figures

**How data were acquired**	Spectral-correlation interferometry (SCI); spectral-phase interferometry (SPI); SCI and SPI biosensors; enzyme linked immunosorbent assay (ELISA); X-ray photoelectron spectroscopy (XPS); proprietary software for SPI; data fitting with commonly used software and a mathematical model that describes adsorption kinetics of molecules on solid phase; statistical t-test.

**Data format**	Raw and analyzed

**Parameters for data collection**	Affordable single-used microscope cover glass slips were used as the biochips. Each stage of the biochip preparation was controlled with X-ray photoelectron spectroscopy. Each stage of the biosensor operation was monitored in real time. Kinetic parameters were calculated by fitting the data with a mathematical model. The biosensor operation was validated by correlation with ELISA. Analytical characteristics of the developed biosensor were compared with those of other advanced methods.

**Description of data collection**	A multiplex microarray biosensor designed for this research recorded in real time sensograms of the biolayer thickness changes in separate recognition spots on a glass biochip. A 50-µL sample of human blood serum containing autoantibodies such as anti-thyroglobulin (anti-TG) and/or anti-thyroid peroxidase (anti-TPO) was diluted and pumped along the biochip for 10 min at room temperature. The respective antigens were pre-immobilized in different sensing spots of the biochip. Using the sensograms, concentration and kinetic constants of autoantibodies were determined at pumping anti-human antibodies or antigens, respectively.

**Data source location**	Moscow, Russia

**Data accessibility**	With the article

**Related research article**	A.V. Orlov, A.V. Pushkarev, S.L. Znoyko, D.O. Novichikhin, V.A. Bragina, B.G. Gorshkov and P.I. Nikitin, Multiplex label-free biosensor for detection of autoantibodies in human serum: tool for new kinetics-based diagnostics of autoimmune diseases. Biosens. Bioelectron. 159 (2020) 112187, doi: 10.1016/j.bios.2020.112187

## Value of the Data

• The data provide a thorough and comprehensive optimization and verification of all stages of a biosensor development and functioning starting from characterization of biochip surface to validation using clinical samples.
• The data can be useful for those who deal with measuring the kinetic characteristics of biochemical reactions in complex mediums containing non-target components, as well as for those who use chemical modifications of glass surfaces in biosensing.
• The data represent a solid basis for further developments of high-precision biosensing systems for early diagnostics of autoimmune diseases.

## 1 Data Description

### 1.1 Calculation of kinetic constants of interaction of autoantibodies on the biochip with free antigen

Kinetic constants of interaction of autoantibodies on the biochip with free antigen [1] were calculated using the commonly applied model for determination of kinetic constants [2]. For this purpose, we used the sensograms recorded by the label-free biosensors based on the spectral-correlation interferometry (SCI) [3,4] and spectral-phase interferometry [5]. The sensogram fragments, which corresponded to the stage of binding of free antigen with the autoantibody on the biochip, were fit with the equation

$$R\left(t\right)=R_{max}\cdot \left(1-exp\left(-k_{ob}\cdot t\right)\right),$$

where *R(t)* – temporal dependence of biolayer thickness, *R_max_* – maximum increment of the biolayer thickness at the stage of antigen binding with autoantibody, *k_ob_* – observed kinetic constant of association. The temporal dependence of biolayer thickness describes a bimolecular reaction between native antigen *Ag* in the sample with autoantibodies *Ab* on the biochip surface:

Ag+Ab↔AgAb.

The antigen concentration *[Ag]_0_* in the solution was maintained constant, and the kinetic constants of association *k_on_* and dissociation *k_off_* were calculated from the equation:

$$k_{ob}=k_{off}+k_{on}\cdot {\left[Ag\right]}_{0\;\;}.$$

## 2 Specificity of the biosensor

### 2.1 Specificity to potentially interfering molecules added to serum samples

We checked the following potentially interfering molecules: i) high-molecular-weight proteins: thyroid stimulating hormone (TSH) and prostate specific antigen; ii) low-molecular-weight substances: biotin (BIO) and chloramphenicol (CAP); iii) mixture of human immunoglobulins (IgG); iv) hepatitis B surface antigen (HBsAg) as a self-assembling protein; v) DNA and RNA molecules.

**Fig. 1:**
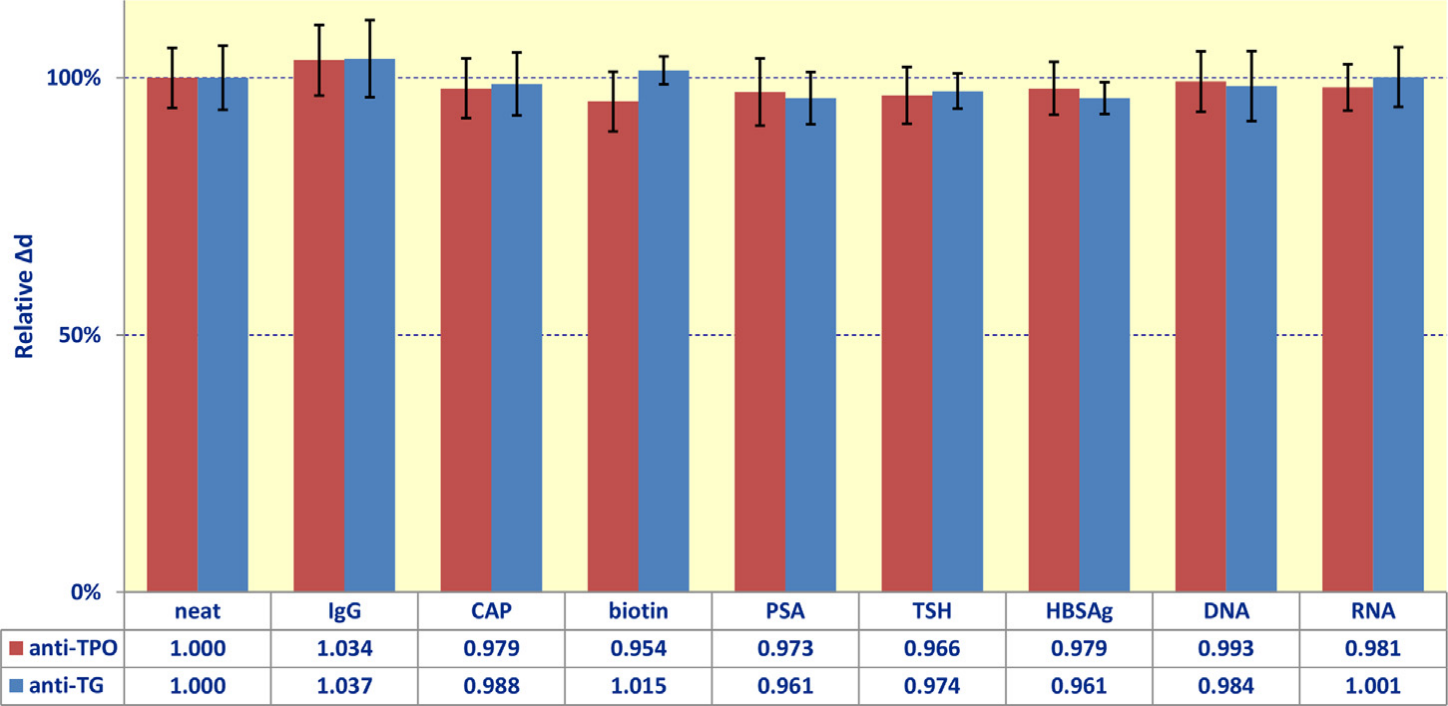
Signals of the developed biosensor upon addition of various potentially interfering molecules to the serum samples (one interfering substance in each experiment): *IgG* – mixture of human immunoglobulins, *CAP* – chloramphenicol, *PSA* - prostate specific antigen, *TSH* - thyroid stimulating hormone, *HBsAg* - hepatitis B surface antigen, *DNA* - deoxyribonucleic acid, *RNA* - ribonucleic acid.

The effect of each individual substance was tested separately by estimation of biomolecular binding [6,7]. The substance (10 IU/mL for TSH; 10 µg/mL for every other substance) was added to the serum samples containing both analyzed autoantibodies: anti-thyroglobulin (anti-TG), concentration of 971 IU/mL and anti-thyroid peroxidase (anti-TPO), concentration of 75 IU/mL. Then we measured the concentrations of anti-TG and anti-TPO. The obtained data can be seen in Fig. 1. In this and other bar plots in this paper, Y-axis shows relative Δd, which is the ratio of measured signal Δd to the specific signal obtained at the standard (initial) conditions for neat positive serum.

### 2.2 Specificity of secondary antibody binding

**Fig. 2:**
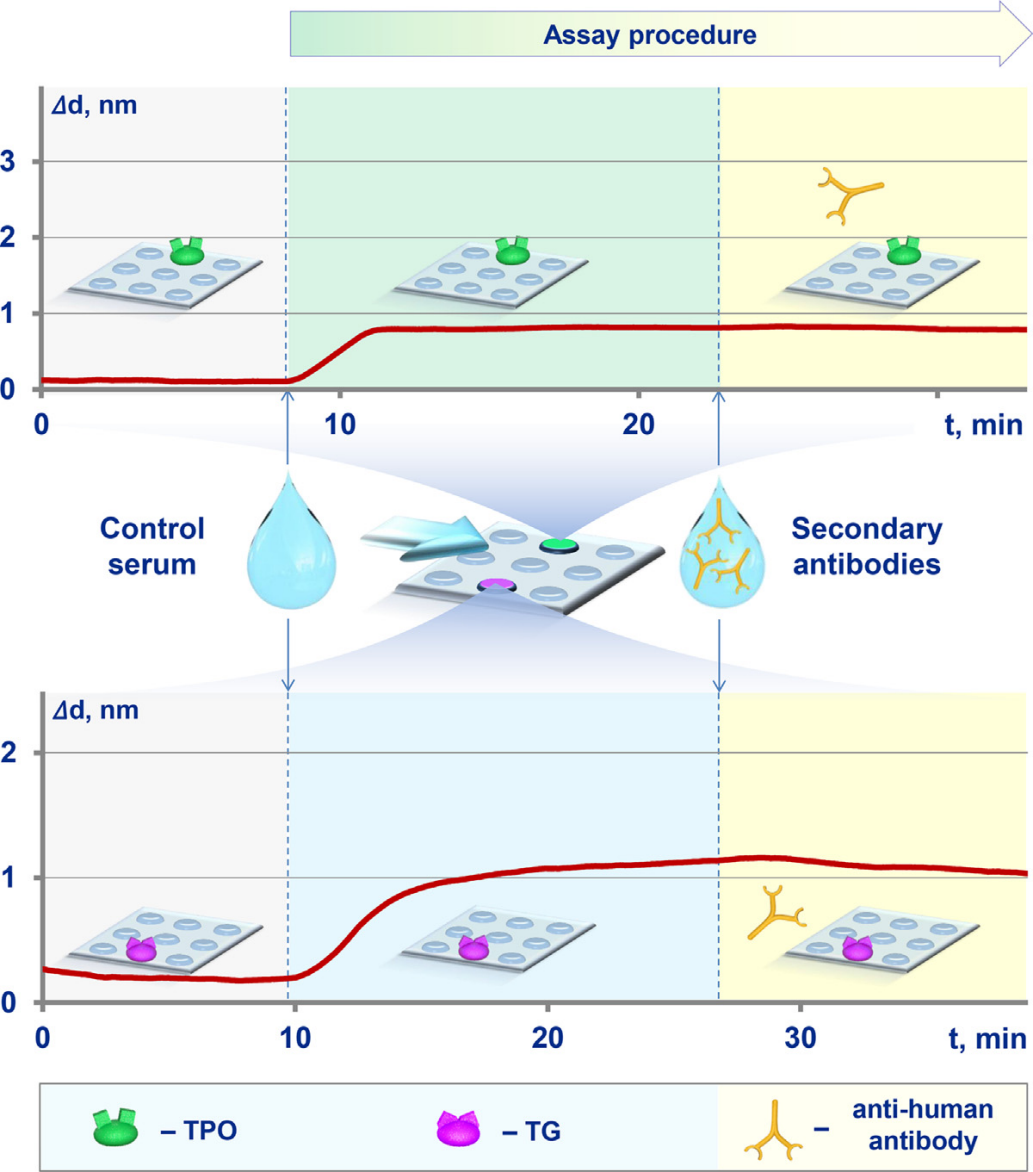
Sensograms of measuring serum samples that contained neither anti-thyroid peroxidase nor anti-thyroglobulin autoantibodies (verification of specific binding of secondary antibodies).

In these experiments, we used serum samples that contained neither anti-thyroid peroxidase nor anti-thyroglobulin autoantibodies. The biolayer thickness increased during pumping such samples along the biochip with immobilized antigens (see characteristic sensograms in Fig. 2). However, at the next stage, when we pumped anti-human antibody that specifically recognized autoantibody-antigen complexes, the biolayer was practically unchanged. The slight decrease in the biolayer thickness at that stage was due to washing out the components that non-specifically immobilized at the previous stage. The obtained data do not exhibit non-specific binding of secondary antibodies.

**Fig. 3:**
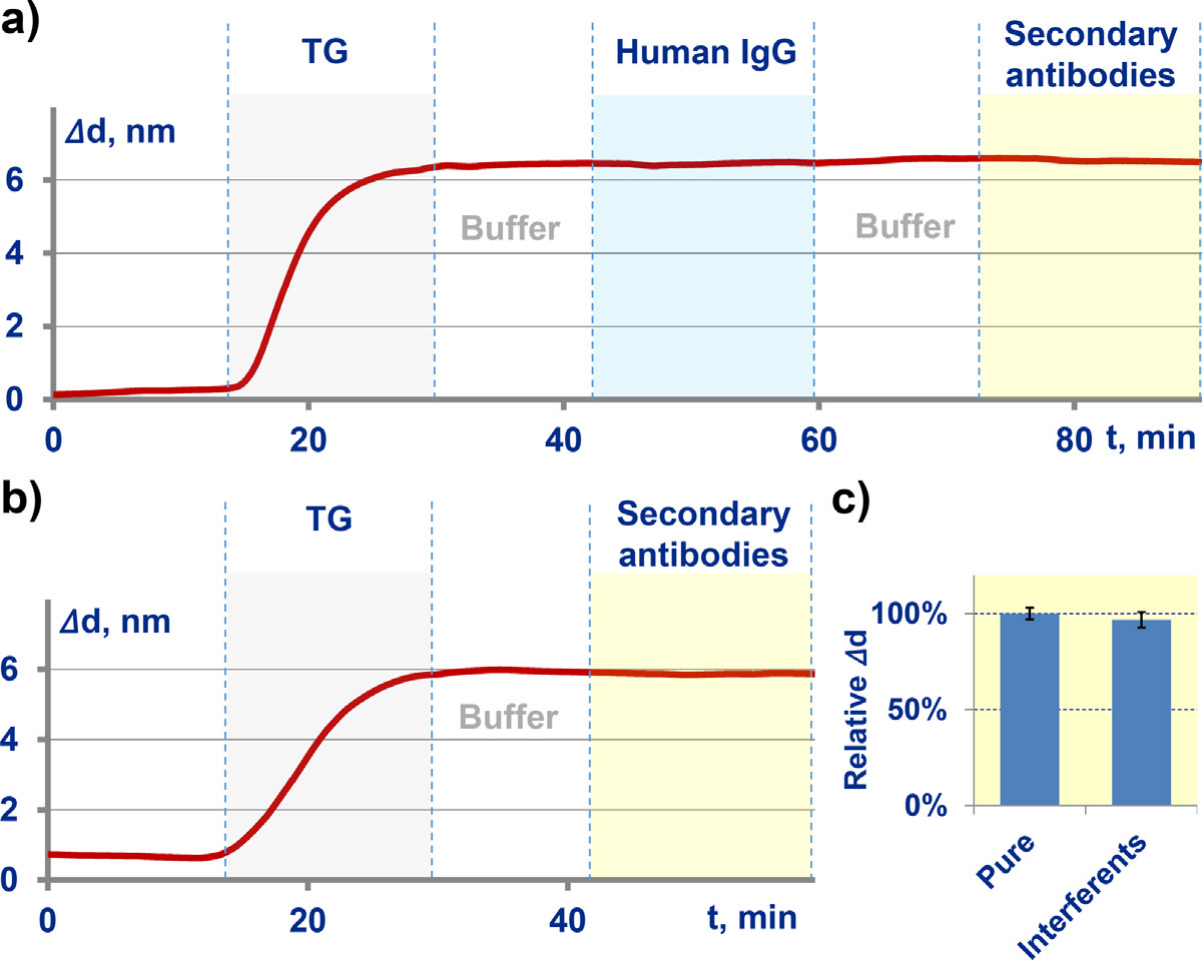
Verification of specific binding of secondary antibodies: a – binding of serum immunoglobulins with antigen on the surface and related binding of secondary antibody; b – binding of secondary antibody with antigen on the surface; с – change in the efficiency of recognizing target immunoglobulins by secondary antibody upon addition of potential interferents.

The absence of immunoglobulins among the non-specific reactants bound to the surface was verified in a modified setup. The analyzed serum was replaced with immunoglobulin fraction of serum. The immunoglobulin concentration of 10 mg/mL was close to that in human blood serum. Free thyroglobulin (20 µg/mL) was added to serum immunoglobulin to block the autoantibodies that may be present. In these experiments, no biolayer increment was observed when pumping the immunoglobulin fraction followed by passing secondary anti-human antibodies (Fig. 3a). The data show no increment in the biolayer thickness due to non-specific binding of secondary antibody with antigen on the surface and no effect of potential interferents on the efficiency of recognition of target immunoglobulins by secondary antibody (Figs. 3b and 3c, respectively).

### 2.3 Specific binding of target antibodies with antibody-antigen complexes

**Fig. 4:**
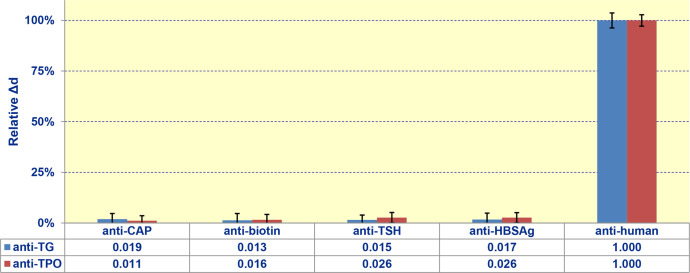
Signals of the developed biosensor in the experiments, in which various non-target antibodies (concentration - 50 μg/mL) were pumped at the stage of passing anti-human antibody. The antibodies tested as non-target: anti-CAP, anti-BIO, anti-TSH, anti-HBsAg.

In these experiments, which were implemented in the single-channel mode of the SPI biosensor [8], various non-target antibodies in concentration 50 μg/mL were pumped instead of anti-human antibody. As non-target antibodies, we tested antibodies to: i) thyroid-stimulating hormone; ii) chloramphenicol; iii) biotin; iv) hepatitis B surface antigen. The data obtained under pumping the non-specific antibodies did not exceed the noise level (Fig. 4).

### 2.4 Verification of absence of interference between immobilized proteins

**Fig. 5:**
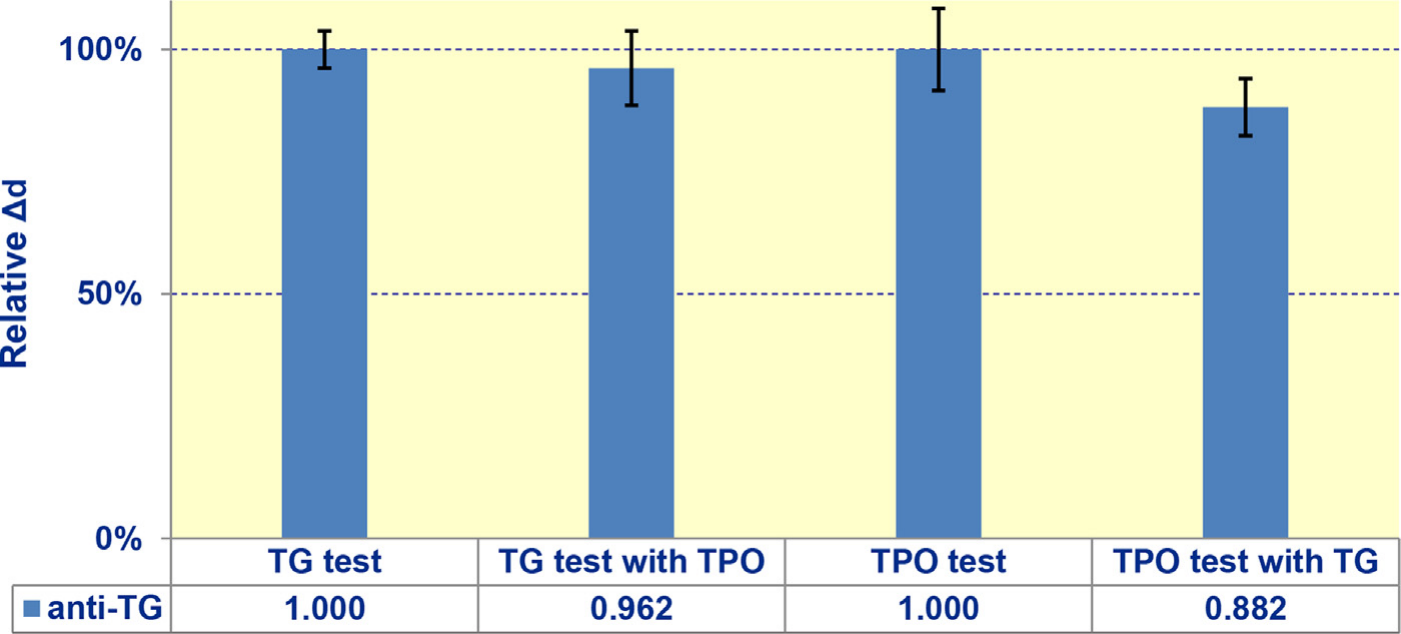
Signals of the developed biosensor while assessment of interference between immobilized proteins.

This experimental series was implemented in the single-channel mode of the biosensor. The serum samples to be tested for anti-TPO were divided into two groups: the first one was measured as usual, while to the other samples, thyroglobulin was added before the measurements. The serum samples to be tested for anti-TG were prepared in the same way by addition of TPO and were measured in the similar setup. The obtained data are exhibited in Fig. 5. The statistical insignificance of differences in the signals was confirmed by p-values of 0.45 and 0.18 calculated for anti-TPO and anti-TG, respectively, both exceeding 0.1.

### 2.5 Specificity of determination of native kinetics

**Fig. 6:**
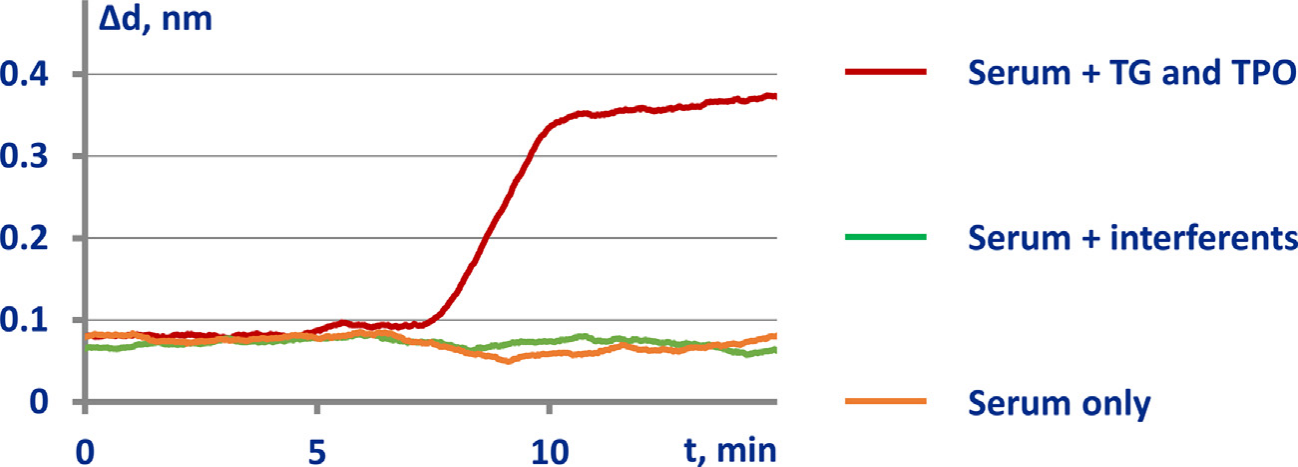
Assessment of specificity of determination of native kinetics.

We compared the data obtained in three series of experiments, which differed in the solutions pumped after passing the serum samples containing anti-TPO and anti-TG autoantibodies. The solutions were as follows: 1) the same serum containing anti-TPO and anti-TG autoantibodies with spiked TG (20 µg/mL) and TPO (20 µg/mL); 2) control - the same serum samples, no additions; 3) the same serum samples with addition of potentially interfering molecules (BIO, mixture of human immunoglobulins, HBsAg, DNA and RNA - each of these in concentration of 10 µg/mL; and 10 IU/mL TSH). According to the sensograms shown in Fig. 6, the biolayer increased only in the first series of experiments, in which the serum containing anti-TPO and anti-TG autoantibodies with spiked TG and TPO was pumped at the final step.

### 2.6 Insignificance of steric effect

The kinetic constants of autoantibodies were compared for the same serum in three variants: undiluted, and diluted 3-fold, and 9-fold. This way, the serum samples had different concentrations of autoantibodies. The concentration of undiluted serum was close to the upper limit of clinically relevant range – 864 IU/mL of anti-TPO. The determined kinetic constant was the same within the experimental error (Table 1). The statistical insignificance of differences in the k_on_ values was confirmed by p-values of 0.48 and 0.61 calculated for 3-fold and 9-fold dilutions, respectively, both exceeding 0.1. The data in Table 1 indicate that the higher the dilution factor (column 1), the lower the biolayer thickness of the native non-immobilized antigen binding Δd at the stage of determination of native kinetics (column 3). Thus, the superficial density of autoantibodies bound to the surface at the previous stage should also decrease with increasing of dilution factor.

**Table 1:** Kinetic constants for the same serum in various dilutions.

Dilution	k_on_ x 10^6^, IU^-1^ x s^-1^ x mL	Δd, nm
Undiluted	94±17	0.43±0.03
3-fold	79±13	0.31±0.02
9-fold	86±15	0.22±0.01

## 3 Optimization of the assay

### 3.1 Chemical modification of glass surface of biochips

The process of chemical modification of the glass biochip surface was optimized toward maximization of the biosensor signal at the final step of the assay [9,10]. Carboxylation and epoxylation were regarded as the candidate techniques. Amination was not considered because it required activation of carboxyl groups on proteins, which might cause conjugation of carboxyl groups with amino groups of the proteins and protein agglomeration.

**Fig. 7:**
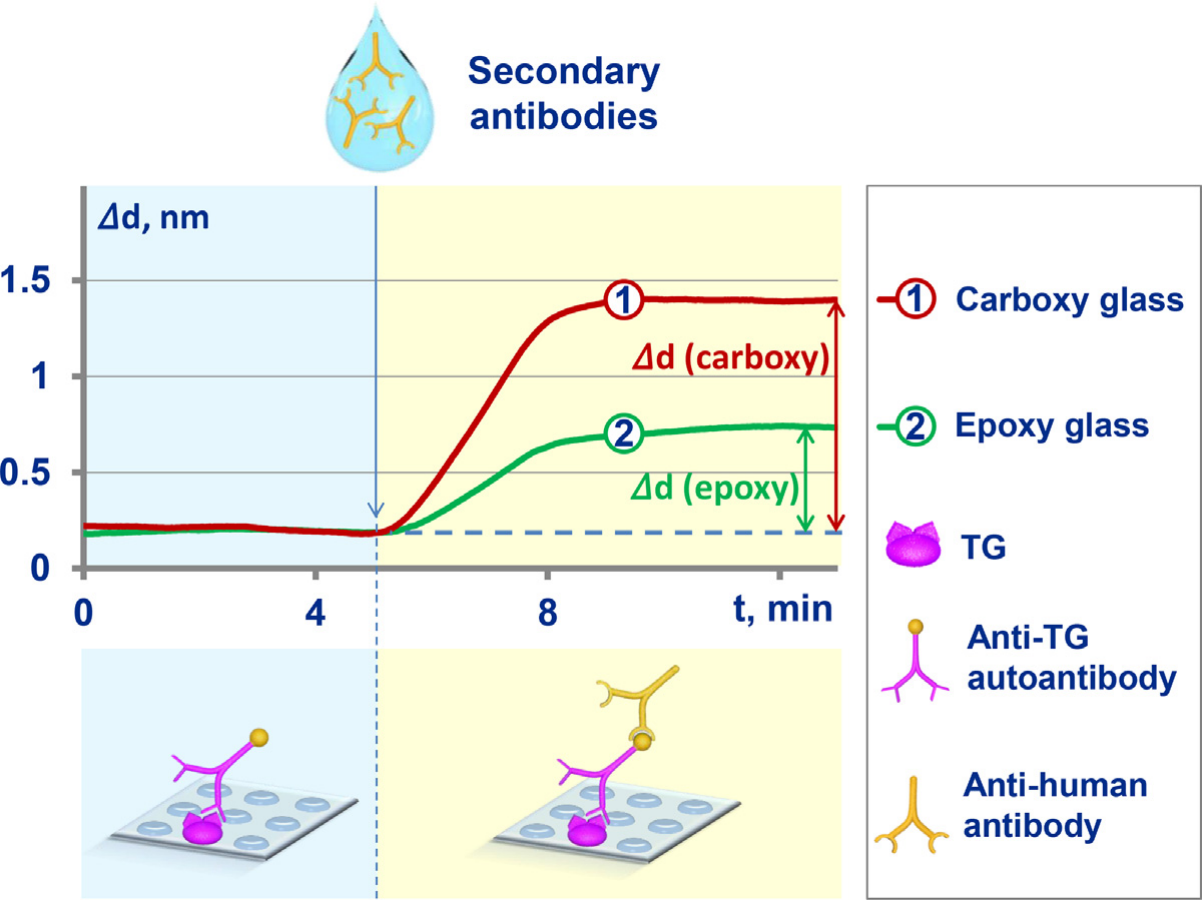
Comparison of increments in the thickness of the secondary antibody biolayer for the epoxylated and carboxylated sensor chips.

The carboxylated and epoxylated glass cover slips were prepared. Onto the obtained slips, thyroglobulin was covalently immobilized directly in the biosensor liquid handling system with additional pre-activation of the carboxylated glass slips by N-(3-Dimethylaminopropyl)-N-ethylcarbodiimide hydrochloride. After that, the surface was blocked with 10 mg/mL of bovine serum albumin (BSA) in phosphate-buffered saline (PBS) buffer followed by pumping of the analyzed serum sample, diluted 10-fold in the same buffer. Each reagent was pumped until adsorption-desorption equilibrium determined by the sensogram plateauing. After the serum, we pumped 10 mg/mL PBS-BSA for a short time and then secondary antibodies diluted in the same buffer. The maximal increase of the biolayer on the sensor chip was measured (Fig. 7).

### 3.2 Antibody screening

**Fig. 8:**
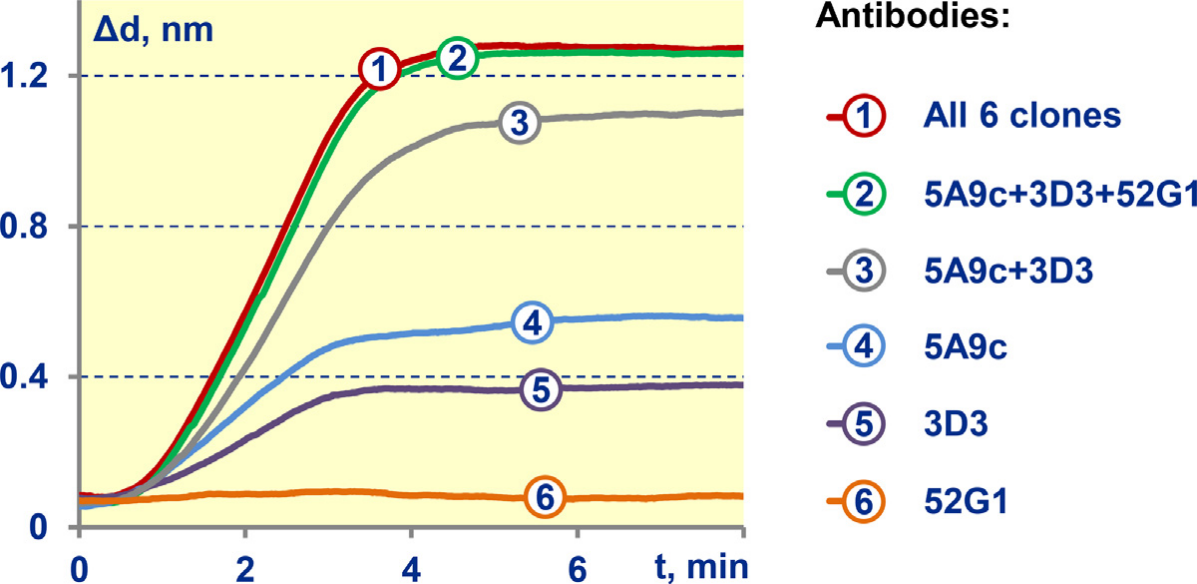
Comparison of the assay sensograms obtained with top six optimal antibody variants.

Another important optimization task was selection of secondary antibody, which was done similarly to the previously reported SCI screening of antibodies for magnetic immunoassays [11,12] interrogated by the frequency mixing technique [13,14]. In the present work, six different clones were screened. We carried out measurements with each single clone, as well as their different combinations in the total concentration of 50 µg/mL. The setup and protocols of these experiments were the same as those given in section 3.1 except changing antibodies instead of surface modification type. Fig. 8 exhibits the sensograms for the optimal variants, while the data for other variants can be found in Table 2.

**Table 2:** Raw data on antibody screening.

Antibody clone	Concentration, µg/mL	Thickness, nm
Polyclone P45	50	0.41 ± 0.03
52G1	50	0.09 ± 0.02
3D3	50	0.32 ± 0.02
5A9C	50	0.48 ± 0.04
5A9C + 3D3	25+25	1.03 ± 0.08
5A9C + 3D3 + 52G1	16.3+16.3+16.3	1.17 ± 0.11
All 6 clones	8.3 for each clone	1.19 ± 0.07
2C11	50	0.05 ± 0.01
5C7	50	0.06 ± 0.01
5G12	50	0.08 ± 0.02

### 3.3 Durations of pumping at each step

To minimize the total assay time, we have optimized the durations of pumping of each reagent along the sensor chip surface. The assay consists of two stages: pumping the analyzed serum sample and pumping the secondary antibody. As the optimal duration, we considered the time between the start of pumping until the sensogram plateauing plus two minutes. These two minutes were added to assure the sensogram plateauing and for more precise determining the biolayer increment value. The optimal duration of serum pumping was found to be 14-18 min (the limiting factor was duration of binding of serum components with the spot where thyroglobulin was immobilized). The time for pumping of secondary antibodies was about 6 min.

### 3.4 Flow rate

**Fig. 9:**
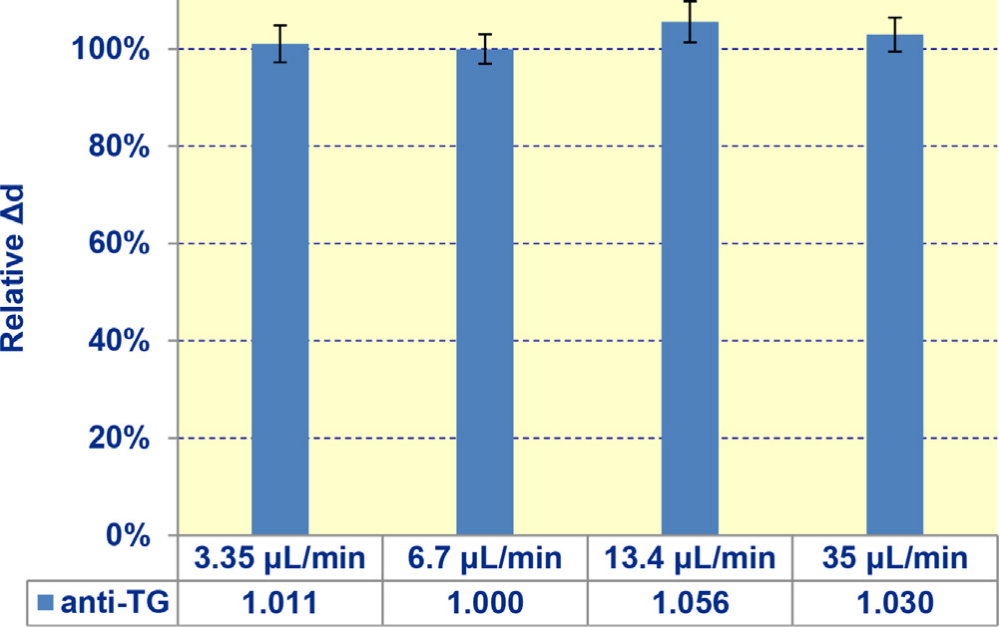
Effect of flow rate on the biosensor signal (signals for anti-TG are shown).

In this experimental series, we measured anti-TG and anti-TPO at: i) low (3.35 μL/min); ii) medium (6.7 μL/min); iii) high (13.4 μL/min) and iv) extremely high 35 μL/min flow rates. The data for anti-TG shown in Fig. 9 were the same within experimental error. The similar data were obtained for anti-TPO.

The possible effect of mass transfer on the procedure of determining the constants (k_ob_, k_on_, k_off_, K_A_, K_D_) was also studied at the flow rates in the range of (3.35 – 35) μL/min. As an example, the values of the observed constant k_ob_ at different flow rates during determination of native kinetic characteristics of binding TG antigen with autoantibodies in the clinical sample of patient #3 are shown in Table 3. The observed constant is the same within experimental error, as well as the constants k_on_, k_off_, K_A_, K_D_ calculated based on the obtained values of k_ob_. That suggests no significant effect of mass transfer limitations. The flow rate of 6.7 μL/min used in the research was optimal in view of the reagent consumption and reliable absence of mass transfer limitations.

**Table 3:** Values of the observed constant k_ob_ at different flow rates

Flow rate	3.35 μL/min	6.7 μL/min	13.4 μL/min	35 μL/min
k_ob_ x 10^3^, s^-1^	16 ± 4	19 ± 5	17 ± 3	22 ± 4

### 3.5 Effect of pH

**Fig. 10:**
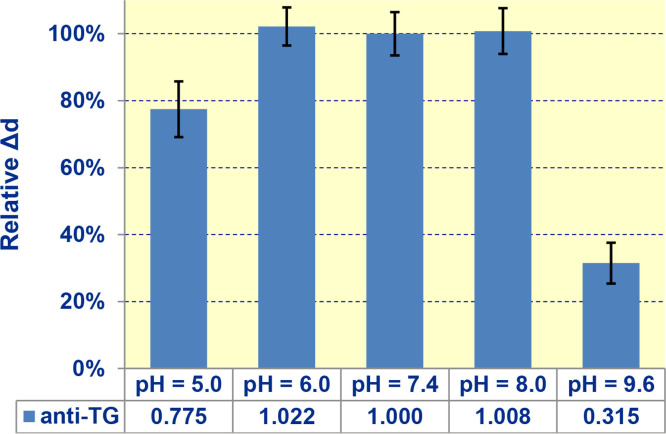
The effect of pH on the biosensor signal.

The effect of buffer pH was investigated using buffers of different pH values: 5.0, 6.0, 7.4, 8.0, 9.6. For the measurements, we added 40 µL of 10 mg/mL BSA diluted in the chosen buffer to 50 µL of human blood serum. The resulting solution was pumped for 10 min at room temperature along the glass biochip having respective antigens pre-immobilized in different sensing spots. At this stage, the IgG autoantibodies in blood serum bound with the antigen on the biochip. Then, 10 mg/mL BSA diluted in the chosen buffer was pumped along the sensor chip. After that, a 50 µg/mL solution of secondary recognition antibody (goat antibody to human immunoglobulins) in 10 mg/mL BSA diluted in the chosen buffer was pumped. The data presented in Fig. 10 show no changes in signal within experimental error in the pH range of 6.0 – 8.0. At pH < 6.0 and pH > 8.0, the signal decreases. In the pH range of 7.35 – 7.45, relevant to blood (serum), in which autoantibodies should be measured, the signal is uniform within experimental error.

## 4 Correlation of the developed biosensor with ELISA

**Fig. 11:**
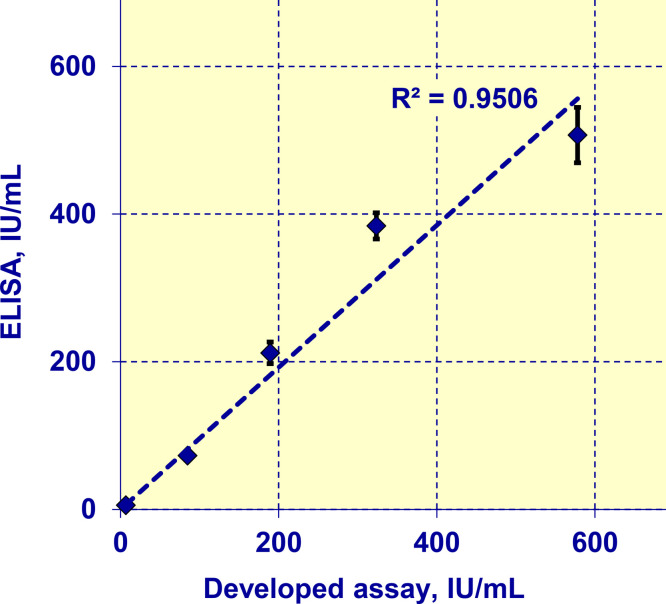
Correlation of the data obtained by the developed multiplex label-free biosensor and ELISA for detection of anti-TG in clinical samples.

We compared the data obtained by measuring the same serum samples by both the proposed assay and ELISA (Table 4 and Fig. 11).

**Table 4:** Raw data on correlation of the developed biosensor with ELISA.

Patient #	Anti-thyroid peroxidase autoantibody		Anti-thyroglobulin autoantibody	
	ELISA, IU/mL	Developed assay, IU/mL	ELISA, IU/mL	Developed assay, IU/mL
1	0.9	1.85	5.8	6.7
2	865	971	73	85
3	1.1	2.33	212	189
4	0.8	0.96	384	324
5	2.4	3.7	507	578

## 5 Comparison of the developed biosensor with traditional methods

Table 5 exhibits the side-by-side comparison of analytical characteristics of the developed biosensor with the most sensitive modern techniques.

**Table 5:** Comparison of analytical characteristics of the developed biosensor for measuring anti-TG and anti-TPO in serum with those of label-based methods

Name (manufacturer)	Labels	Detection method	LOD for anti-TPO, IU/mL	LOD for anti-TG, IU/mL	Simultaneous detection of several autoantibodies in a single sample	Determination of kinetic characteristics	Duration, min	Refs
The presented biosensor	Label-free	Multiplex spectral-correlation interferometry	1.7	6	Yes	Yes	25	[1]
RIA kits (Institute of isotopes Ltd.)	^125^I (radioactive)	Radioimmunometric	2	13	No	No	180	[15,16]
RIA kits (Demeditec Diagnostics GmbH)	^125^I (radioactive)	Radioimmunometric	2.4	8.6	No	No	120-180	[17,18]
DYNOtest (BRAHMS Diagnostica)	^125^I (radioactive)	Radioimmunometric	5.5	5.5	No	No	180	[19], [20], [21]
ELISA kits (Demeditec Diagnostics GmbH)	Horseradish peroxidase	Colorimetric	5	10	No	No	65	[22,23]
AccuBind® ELISA Kits (Monobind Inc.)	Horseradish peroxidase	Colorimetric	1.5	5	No	No	105	[24,25]
Elecsys® (Roche Diagnostics)	Ruthenium	Electrochemiluminescence	5	10	No	No	20	[20,26,27]
ARCHITECT i2000 (Abbott Diagnostics)	Acridinium esters	Bead-based chemiluminescence	1	1	No	No	29	[28], [29], [30]
ADVIA Centaur®, (Siemens Healthineer)	Acridinium esters	Bead-based chemiluminescence	15	10	No	No	18	[28], [29], [30], [31]
TRACE Kryptor, Brahms Diagnostica	Europium cryptate/ XL 665	Fluorescence	1.8	10	No	No	29	[27,31,32]

**Fig. 12:**
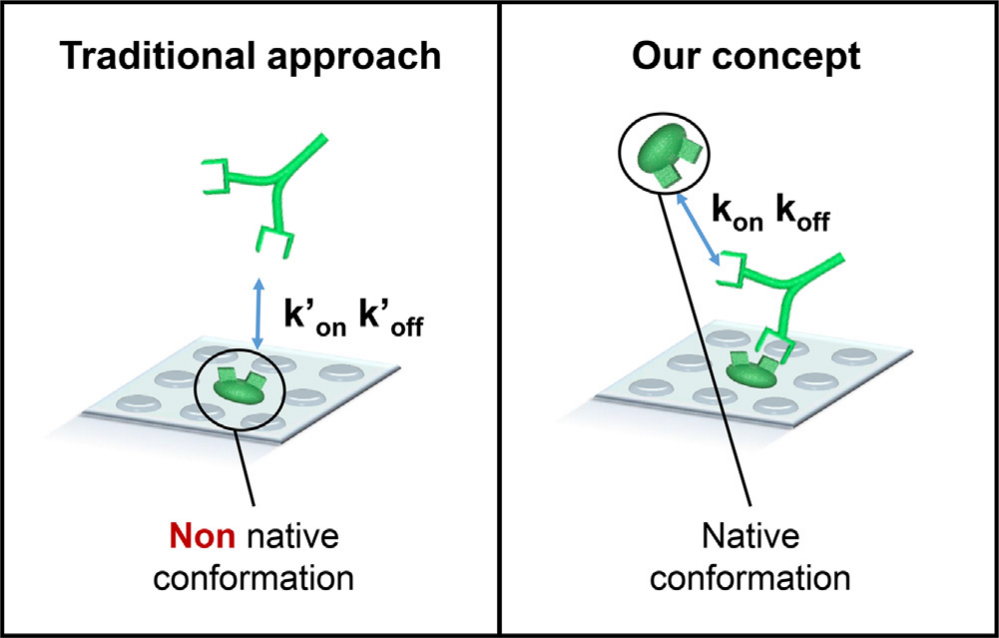
Difference between our principle and traditional label-free approaches for determination of kinetic parameters of autoantibody in serum.

Fig. 12 illustrates the difference between our principle and traditional label-free techniques. Our principle based on autoantibody polyvalency has permitted, for the first time, registration of autoantibody interaction with free antigens in serum rather than those immobilized on a surface. Our approach allows avoiding most of the surface-induced issues (described in the related Biosens. Bioelectron. paper [1]), which are due to unpredictable conformation of surface-deposited antigens.

## 6 Linearity of dilution and recovery test

Since all experiments with autoantibodies are carried out in clinical serum, “spike and recovery tests” are not appropriate. We carried out the “linearity of dilution” test, and the obtained concentration data are given in Table 6.

**Table 6:** Linearity of dilution test.

anti-TPO concentration				anti-TG concentration			
Serum from patient #2				Serum from patient #5			
Dilution factor	Measured, IU/mL	Expected, IU/mL	% Expected	Dilution factor	Measured, IU/mL	Expected, IU/mL	% Expected
1	971	–	–	1	578	–	–
2	507.3	485.5	104.5%	2	284.2	289.0	98.3%
4	237.9	242.8	98.0%	4	136.4	144.5	94.4%
8	113.5	121.4	93.5%	8	76.9	72.3	106.4%
16	61.1	60.7	100.8%	16	37.6	36.1	104.1%

## 7 Biochip characterization

### 7.1 Mechanism of antigen attachment to the biochip surface

**Fig. 13:**
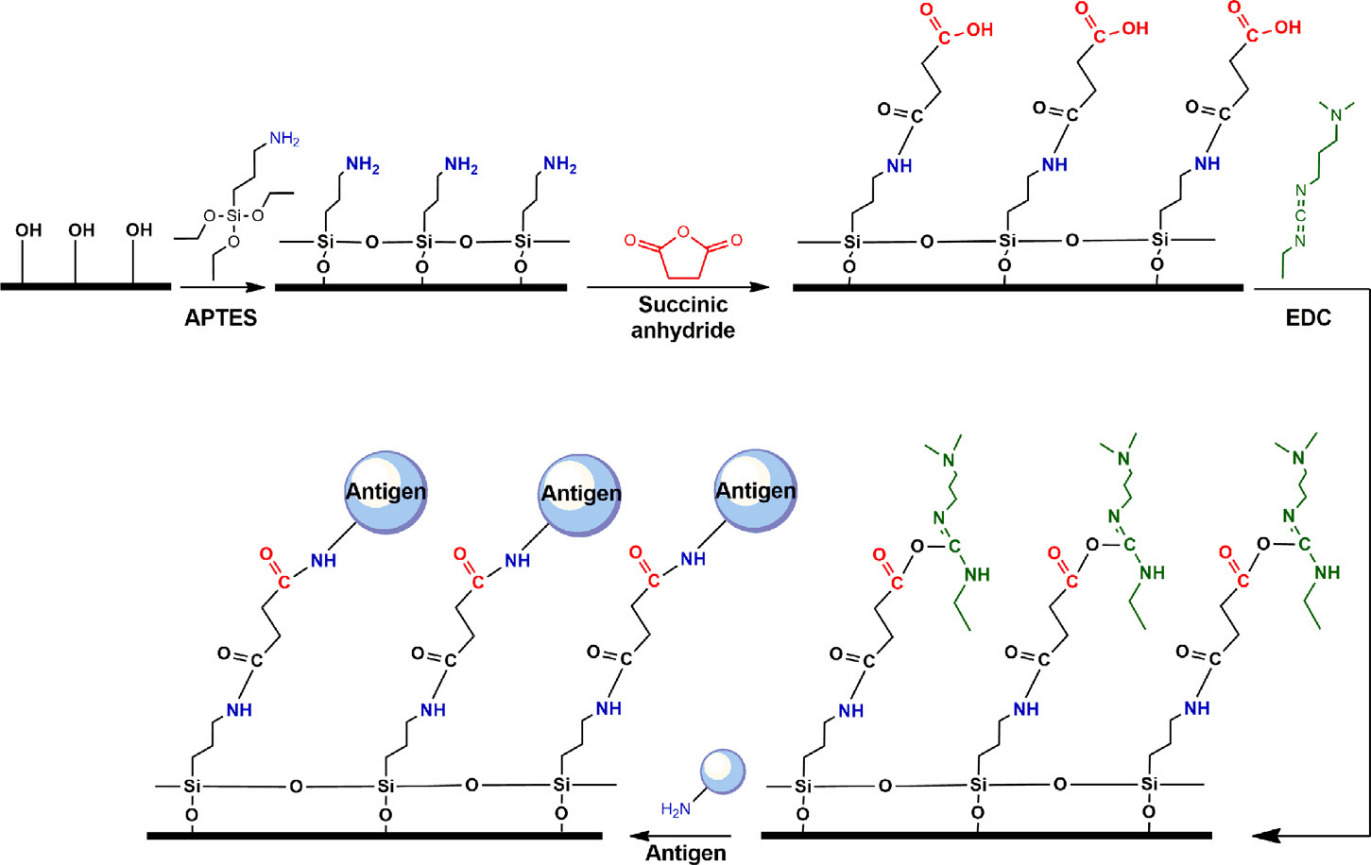
Mechanism of antigen attachment onto the glass surface.

The mechanism of antigen attachment to the biochip glass surface is illustrated in Fig. 13 (see also the full protocol in section 2.3 of the related Biosens. Bioelectron. paper [1]). The glass surface pre-treated with piranha solution (Fig. 13a) is modified with aminosylane (APTES) to introduce an amino group (NH_2_) as a basis for the following functionalization (Fig. 13b). Further treatment with succinic anhydride (SA) forms a carboxyl group (COOH) on the surface (Fig. 13c) that is activated at the next step with carbodiimide (EDC) to produce o-acylisourea active ester (Fig. 13d). The latter reacts with an amino group (NH_2_) of antigen with the formation of a covalent peptide bond (Fig. 13e).

### 7.2 Instrumental control of chemical modifications of biochip surface

**Fig. 14:**
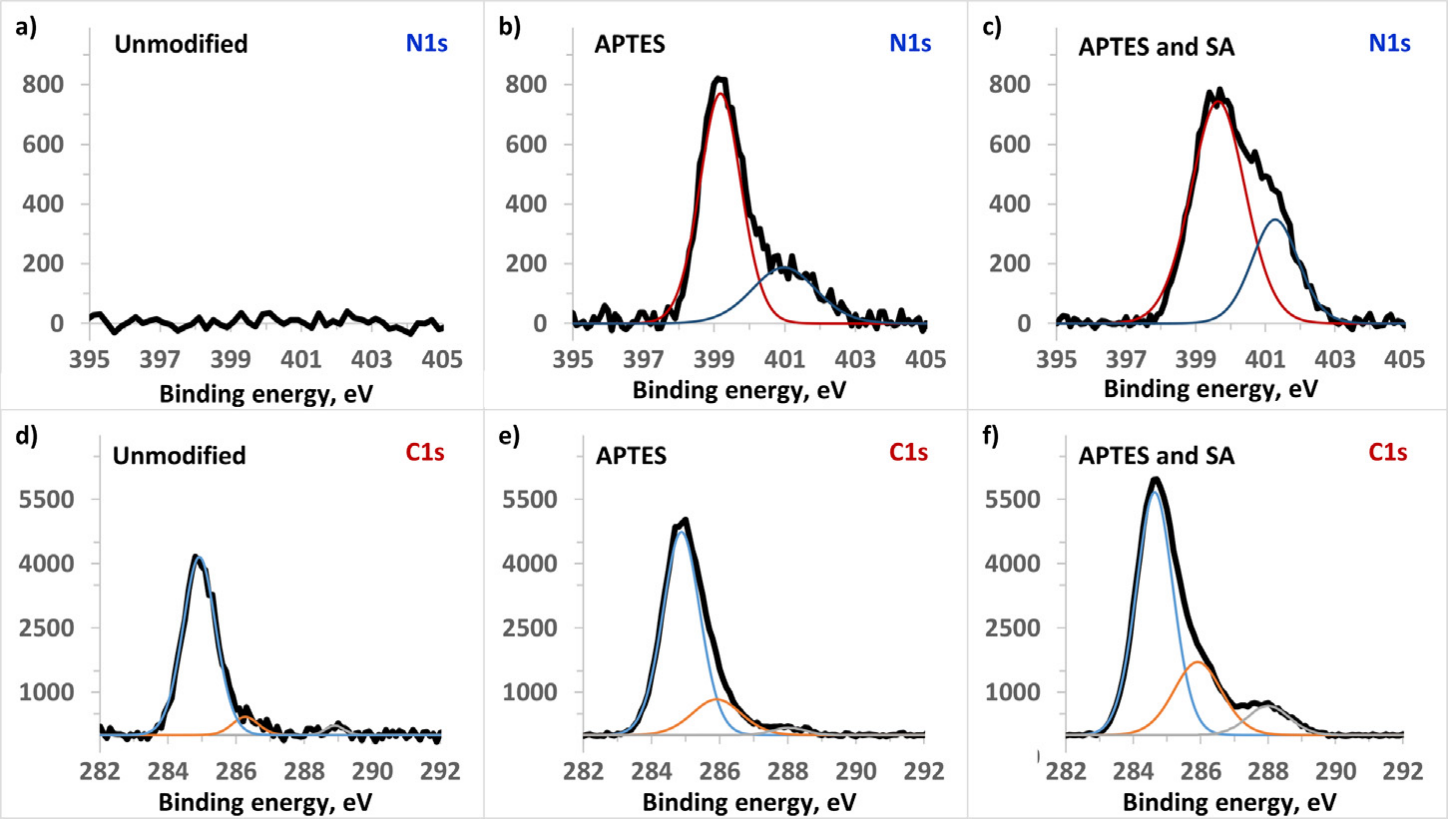
XPS-spectra of the biochip surface: *a, d* – before modification; *b, e* – after modification with APTES; *c, f* – after modification with succinic anhydride.

The process of chemical modifications of the slip during fabrication of the biochip was controlled with X-ray photoelectron spectroscopy (XPS). For this purpose, we employed the photoelectron spectrometer “KRATOS AXIS ULTRA DLD” with spherical analyzer, ion gun, UV- and X-ray sources. Charge neutralization was used for all samples. The binding energy scale was charge referenced to the C1s at 284.7 eV. The characteristic peaks in the spectra to be analyzed are as follows: nitrogen N1s - at 400 eV (see Fig. 14a, b, c), carbon C1s – at 286 eV and 289 eV for С-О and С=О bonds, respectively (Fig. 14 d, e, f).

The spectrum of an original, non-modified glass is shown in Fig. 14a. The Figs. 14b, c show pronounced peaks at 400 eV, corresponding to amino groups appeared on the surface after incubation with APTES. The carboxylated glass produced by subsequent incubation with succinic anhydride (Fig. 14f), in contrast to the aminated and unmodified ones (Figs. 14d, e), exhibits a peak at 288 eV that corresponds to С=О bond, which is available in carboxyl group and amide bond. The peak at 286 eV appears in the spectra of both aminated and carboxylated surfaces. That peak corresponds to С-О bond, which presents in carboxyl group and is formed during silanization of the surface.

### 7.3 Biochip activation

**Fig. 15:**
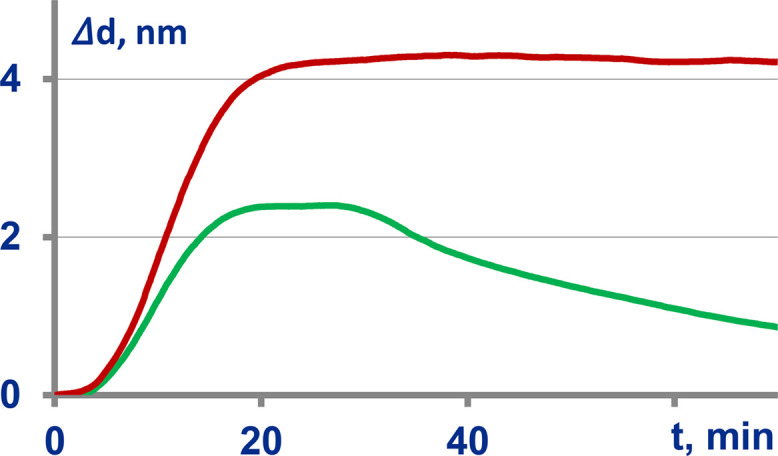
Sensograms of stable immobilization of proteins on the fabricated surface after its activation with carbodiimide (*top,* red line) and reversible immobilization on the unactivated surface (*bottom,* green line) with desorption after pumping PBS-BSA buffer at 30 min.

The effect of biochip activation on stability of protein immobilization was assessed as follows. The immobilization process was real-time monitored with non-activated biochips and those activated with carbodiimide. The characteristic sensograms recorded in these experiments are shown in Fig. 15.

### 7.4 Biochip shelf-life

**Fig. 16:**
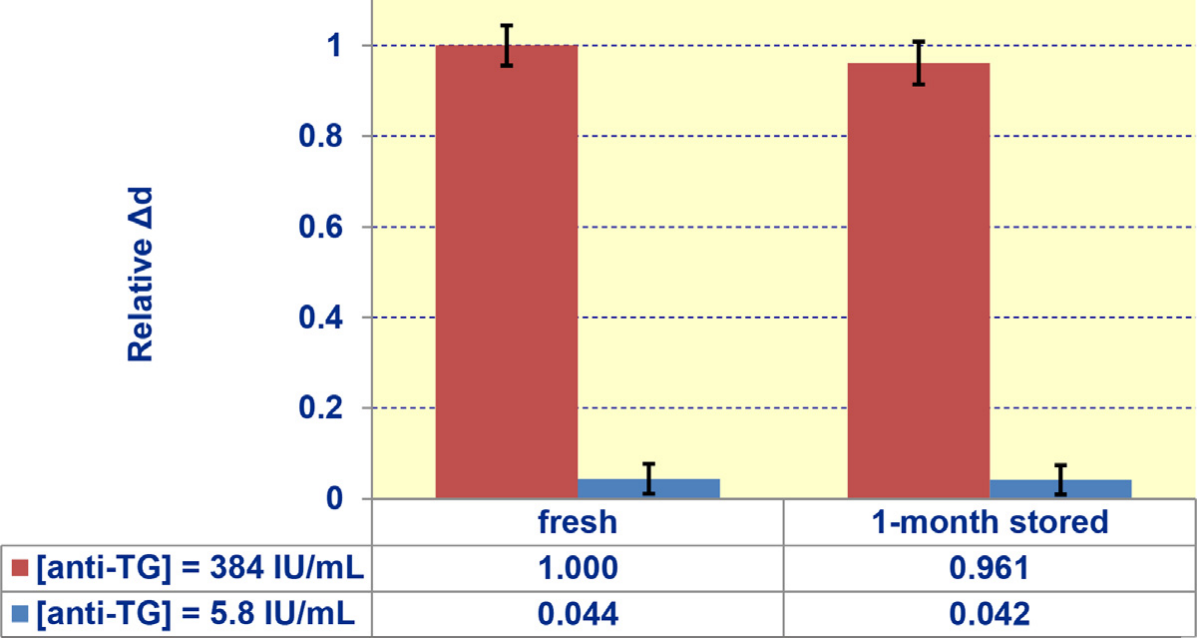
Comparison of the signals obtained with serums of low (5.8 IU/mL, *blue* bars) and high (384 IU/mL, *red* bars) anti-TG levels using "fresh" and one-month stored biochips.

Here, the immobilization stage was implemented inside the biosensor to demonstrate real-time registration of all reactions. This stage, though, is not required for the biosensor operation. The biochips can be prepared in advance. To estimate the shelf-life of the biochips, the immobilization was done by spot deposition rather than inside the biosensor, and was followed by drying at room temperature and further refrigerating at +4 ^o^C. After one-month storage, the biochips were used in the experiments. Fig. 16 shows similar (within experimental error) signals obtained with "fresh" and stored biochips. The statistical insignificance of differences in the signals is confirmed by p-value of 0.44 exceeding 0.1.

## 8 Raw data from the sensor

Table 7 presents the fragments of raw data of sensograms obtained for both native kinetics determination and concentration measurement of different autoantibodies in the same serum sample.

**Table 7:** Raw data recorded by the developed biosensor for simultaneous detection and kinetics characterization of different autoantibodies in the same serum sample.

Time, min	Δd, nm				Time, min	Δd, nm			
	Native kinetics determination		Concentration measurement			Native kinetics determination		Concentration measurement	
	TPO	TG	TPO	TG		TPO	TG	TPO	TG
25.02448	1.82528	1.34450	2.10271	1.09608	28.92435	1.90588	1.46356	2.90177	1.98803
25.07331	1.82791	1.34472	2.10401	1.09731	28.97318	1.90586	1.46665	2.90250	1.99066
25.12214	1.83063	1.34571	2.10492	1.10049	29.02188	1.90713	1.46916	2.90928	1.99355
25.17096	1.83227	1.34599	2.10359	1.10081	29.07071	1.90932	1.47217	2.91324	1.99403
25.21979	1.83374	1.34501	2.10622	1.10173	29.11953	1.91329	1.47611	2.91699	1.99364
25.26849	1.83406	1.34443	2.10596	1.10240	29.16823	1.91639	1.47938	2.91607	1.99817
25.31719	1.83395	1.34427	2.10733	1.10111	29.21706	1.92195	1.48280	2.92024	1.99817
25.36614	1.83463	1.34443	2.10808	1.10213	29.26576	1.92790	1.48623	2.92228	1.99835
25.41484	1.83541	1.34563	2.10635	1.10249	29.31458	1.93173	1.48930	2.92473	1.99823
25.46354	1.83705	1.34575	2.10489	1.10220	29.36342	1.93774	1.49200	2.92687	1.99738
25.51238	1.83794	1.34477	2.09804	1.10047	29.41211	1.94260	1.49457	2.92956	1.99679
25.56108	1.83855	1.34358	2.09683	1.09965	29.46094	1.94992	1.49853	2.93047	1.99580
25.61003	1.83807	1.34328	2.09623	1.10021	29.50964	1.95502	1.50168	2.93173	1.99679
25.65859	1.83596	1.34454	2.09634	1.10076	29.55846	1.96251	1.50467	2.93390	1.99489
25.70756	1.83735	1.34395	2.09844	1.09906	29.60716	1.97066	1.50581	2.93624	1.99585
25.75625	1.83496	1.34467	2.10086	1.10123	29.65599	1.97975	1.50775	2.93586	1.99553
25.80495	1.83783	1.34476	2.10109	1.10189	29.70469	1.98776	1.51129	2.93852	1.99449
25.85378	1.84070	1.34387	2.10143	1.10289	29.75352	1.99639	1.51537	2.94042	1.98936
25.90248	1.84263	1.34401	2.10208	1.10304	29.80234	2.00737	1.51828	2.94064	1.98914
25.95131	1.84565	1.34446	2.10290	1.10522	29.85104	2.01576	1.52118	2.94278	1.98808
26.00013	1.84442	1.34491	2.10232	1.10943	29.89974	2.02317	1.52277	2.94352	1.98767
26.04883	1.84330	1.34506	2.09996	1.11389	29.94870	2.02965	1.52311	2.94302	1.98718
26.09766	1.84619	1.34571	2.09594	1.12049	29.99753	2.04152	1.52477	2.94462	1.98800
26.14636	1.84482	1.34656	2.09046	1.12498	30.04623	2.05224	1.52742	2.94645	1.98628
26.19518	1.84538	1.34813	2.08630	1.13194	30.09493	2.06094	1.53077	2.94558	1.98673
26.24388	1.84400	1.34802	2.08436	1.14146	30.14375	2.06902	1.53276	2.94490	1.98513
26.29284	1.84516	1.34871	2.08045	1.15264	30.19245	2.07966	1.53355	2.94750	1.98338
26.34141	1.84351	1.34856	2.07832	1.16438	30.24128	2.08966	1.53602	2.94696	1.98302
26.39037	1.84165	1.34829	2.07927	1.17902	30.29011	2.09940	1.53611	2.94801	1.98429
26.43893	1.84123	1.34888	2.08031	1.19462	30.33881	2.10914	1.53677	2.94924	1.98406
26.48776	1.84331	1.35051	2.08370	1.21011	30.38763	2.11785	1.53822	2.95151	1.98262
26.53659	1.84498	1.35187	2.08919	1.22852	30.43633	2.12446	1.53928	2.95132	1.98400
26.58529	1.84687	1.35205	2.09648	1.24704	30.48516	2.12874	1.54001	2.95240	1.98541
26.63412	1.84709	1.35127	2.10703	1.26704	30.53386	2.13532	1.54082	2.95253	1.98675
26.68294	1.84574	1.35139	2.11932	1.28896	30.58268	2.13959	1.54279	2.95410	1.98772
26.73164	1.84562	1.35135	2.13524	1.31442	30.63151	2.14594	1.54540	2.95317	1.98980
26.78047	1.84908	1.35183	2.14948	1.33991	30.68021	2.14747	1.54800	2.95427	1.99039
26.82929	1.85194	1.35339	2.16766	1.36527	30.72891	2.15152	1.55045	2.95328	1.99091
26.87799	1.85469	1.35311	2.18677	1.39346	30.77774	2.15166	1.55170	2.95225	1.99209
26.92683	1.85645	1.35442	2.20821	1.42137	30.82656	2.15398	1.55331	2.95124	1.99115
26.97553	1.86096	1.35455	2.22970	1.44813	30.87526	2.15603	1.55391	2.95294	1.99052
27.02435	1.86113	1.35562	2.25545	1.47570	30.92423	2.15893	1.55366	2.95139	1.99262
27.07318	1.85967	1.35633	2.28098	1.50412	30.97292	2.16021	1.55394	2.95074	1.99539
27.12188	1.86016	1.35750	2.30518	1.53224	31.02162	2.16466	1.55451	2.94828	1.99396
27.17070	1.85912	1.35870	2.32830	1.56062	31.07044	2.16699	1.55411	2.94898	1.99326
27.21953	1.86070	1.35909	2.35483	1.58841	31.11914	2.16799	1.55463	2.94937	1.99299
27.26823	1.86333	1.36054	2.38615	1.61566	31.16798	2.16930	1.55540	2.95162	1.99153
27.31706	1.86546	1.36121	2.41839	1.64096	31.21679	2.16716	1.55599	2.95166	1.99306
27.37696	1.87017	1.36326	2.45041	1.66932	31.26563	2.16858	1.55502	2.95053	1.99423
27.43673	1.87132	1.36537	2.48269	1.69490	31.31433	2.16844	1.55574	2.95122	1.99349
27.48542	1.87359	1.36870	2.51367	1.71882	31.36316	2.17046	1.55627	2.95062	1.99542
27.53438	1.87220	1.37126	2.54815	1.74356	31.41185	2.17304	1.55737	2.95045	1.99666
27.58308	1.87501	1.37268	2.58037	1.76563	31.46068	2.17287	1.55861	2.95010	1.99502
27.63178	1.87928	1.37404	2.60997	1.78499	31.50938	2.17352	1.55942	2.94859	1.99584
27.68060	1.88218	1.37773	2.63836	1.80442	31.55821	2.17387	1.55957	2.94834	1.99672
27.72956	1.88250	1.38107	2.66454	1.82275	31.60691	2.17394	1.56048	2.94895	1.99444
27.77813	1.88284	1.38349	2.68547	1.83874	31.65573	2.17285	1.56181	2.94730	1.99251
27.82696	1.88322	1.38650	2.70745	1.85535	31.70456	2.17417	1.56291	2.94515	1.99096
27.87566	1.88297	1.38820	2.72708	1.87085	31.75326	2.17539	1.56320	2.94416	1.98945
27.92448	1.88464	1.39138	2.74655	1.88252	31.80208	2.17674	1.56378	2.94278	1.99028
27.98542	1.88546	1.39539	2.76105	1.89331	31.85091	2.17534	1.56378	2.94326	1.98809
28.04636	1.88470	1.40010	2.77629	1.90422	31.89961	2.17631	1.56344	2.94319	1.98876
28.09518	1.88619	1.40303	2.78858	1.91395	31.94844	2.17674	1.56330	2.94269	1.98946
28.14401	1.88813	1.40607	2.79921	1.92184	31.99714	2.17844	1.56376	2.94334	1.99113
28.19271	1.88928	1.40918	2.80975	1.92978	32.04596	2.17942	1.56561	2.94435	1.99407
28.24141	1.88917	1.41154	2.81945	1.93761	32.09466	2.17712	1.56687	2.94385	1.99467
28.29037	1.89109	1.41384	2.82892	1.94373	32.15456	2.17875	1.56732	2.94584	1.99205
28.33906	1.89507	1.41803	2.83655	1.95028	32.21446	2.18100	1.56687	2.94636	1.99180
28.38789	1.89444	1.42092	2.84526	1.95479	32.26316	2.17808	1.56865	2.94761	1.99283
28.43659	1.89685	1.42472	2.85443	1.96022	32.31198	2.17841	1.56863	2.94629	1.99589
28.48542	1.89882	1.42826	2.86092	1.96532	32.36068	2.17822	1.57085	2.94799	1.99836
28.53412	1.89960	1.43188	2.86534	1.97270	32.40964	2.18002	1.57279	2.94824	1.99910
28.58294	1.89642	1.43602	2.87232	1.97848	32.45833	2.18196	1.57431	2.94864	1.99868
28.63178	1.89650	1.44130	2.87840	1.98045	32.50703	2.18047	1.57432	2.94925	1.99877
28.68048	1.89928	1.44448	2.88329	1.98239	32.55586	2.18266	1.57478	2.94932	1.99768
28.72917	1.90233	1.44842	2.88854	1.98678	32.60469	2.18035	1.57506	2.94970	1.99639
28.77787	1.90295	1.45138	2.88974	1.98371	32.65352	2.17564	1.57664	2.94946	1.99792
28.82683	1.90279	1.45569	2.89302	1.98377	32.70221	2.17351	1.57769	2.94990	1.99693
28.87566	1.90316	1.45971	2.89907	1.98709	32.75091	2.17252	1.57957	2.95120	1.99541

## 9 Experimental Design, Materials, and Methods

Multiplex biosensor based on the spectral-correlation interferometry that uses a microscope cover glass slip as a biochip, affordable to be single-used in medical applications, has been designed. The microarray imaging label-free biosensor employs radiation from a broadband superluminescent diode (SLD-381-MP, Superlum Diodes, Ltd., Russia) in an optical scheme that comprises two interferometers. The base (inter-mirror distance) of one Fabry-Perot interferometer is periodically changed with a piezoelectric driver. The mentioned glass biochip serves simultaneously as the second two-beam reflective interferometer. During biochemical reactions on the biochip, the optical thickness of the slip with the bound biological layer changes. To measure such changes, the interference between a reference beam reflected from the bottom surface of the slip and a beam reflected from the "biolayer – analyzed sample" interface is used. Variations in optical thickness of the biomolecule layer on the recognition spots on the biochip are calculated by phase changes of correlation signals from a 12-bit monochrome CCD-camera (Basler, Germany). For simultaneous registration of biochemical reactions in several recognition spots, their image signals are averaged over the area of each recognition spot.

The following reagents were used in this work: microscope cover glasses, bovine serum albumin (BSA), (3-Aminopropyl)triethoxysilane (APTES), N-(3-Dimethylaminopropyl)-N-ethylcarbodiimide hydrochloride (EDC) (Sigma Aldrich, USA); sulphuric acid, methanol, dimethylformamide (Chimmed, Russia); thyreoperoxidase, thyroglobulin, goat monoclonal antibodies against human IgG (Xema medica, Russia; GeneTex, USA; Russian Research Center for Molecular Diagnostics and Therapy). The serum standards and samples from patients were provided by the Russian Cardiology Research and Production Complex of Russian Ministry of Health (Moscow, Russia), and the present experiments were approved by the ethics committee of this institution. Immunoglobulin fraction of serum for intravenous use was purchased in a local pharmacy.

As the low-cost single-used biochips, microscope glass cover slips that do not require any additional metal or dielectric coatings were used. The biochip surface was carboxilated for efficient and rapid immobilization of biomolecules. For this purpose, the thoroughly cleaned microscope cover slips were incubated for 16 h under an exhaust hood in 3% solution of APTES in methanol and then for 2 h in 15 mM solution of succinic anhydride in dimethylformamide. Then the glasses were thermally processed in a dry heat oven at 105 ºC for 1 h with further washing at room temperature. The chips were activated by 15-min incubation in 20 mg/mL solution of EDC in MES. Then 50 µg/mL of antigens in PBS was deposited in different sensing spots to achieve covalent immobilization of antigen onto the biochip due to interaction of antigen amino groups with activated carboxyl groups on the surface. The surface was blocked with a mixture of 1% glycine and 0.5% of bovine serum albumin (BSA) in PBS buffer. The prepared biochips can be stored without deterioration for a long time until using. Quality control of immunoreagents was implemented as described in Ref. [33].

The process of chemical modifications of the slip during fabrication of the biochip was controlled with X-ray photoelectron spectroscopy (XPS). For this purpose, we employed the photoelectron spectrometer “KRATOS AXIS ULTRA DLD” with spherical analyzer, ion gun, UV- and X-ray sources described in section 7 above.

All experiments in this research were carried out in human blood serum. Each of the samples contained various amounts of anti-TG and anti-TPO, naturally produced by the immune system of the patients (not spiked). 50 µL of the human blood serum was diluted with 40 µL of 10 mg/mL of PBS-BSA and then pumped for 10 min at room temperature along the glass biochip having respective antigens pre-immobilized in different sensing spots. At this stage, some of the IgG autoantibodies in blood serum, which had two binding fragments (Fab), bound with corresponding antigen on the surface with one Fab. The other free Fab was still capable to interact with free antigen. To provide the unique information on autoantibody binding with native antigen, which was not bound to the surface, the same serum samples with the spiked free antigen (both TPO and TG) were pumped. The kinetic constants of autoantibody interaction with native antigen were calculated using the sensograms obtained in these experiments and the model described in section 1 above.

Other procedures and protocols are described in details in Ref. [1].

## Declaration of Competing Interest

P.I.N. and B.G.G. are the named inventors on SCI- and SPI-related patents.
